# Bangladesh HEARTS: Lessons Learned from a National Hypertension Control Program, 2018–2026

**DOI:** 10.5334/gh.1595

**Published:** 2026-10-21

**Authors:** Shamim Jubayer, Md. Ahsan-uz-Zaman, Syed Zakir Hossain, Mahfuzur Rahman Bhuiyan, Obehioye Aimiosior, Ahmad Abrar, Rahat Iqbal Chowdhury, Mohammad Robed Amin, Bolanle F. Banigbe, Reena Gupta, Andrew E. Moran, Thomas R. Frieden, SohelReza Choudhury

**Affiliations:** 1 National Heart Foundation Hospital and Research Institute, Dhaka, Bangladesh; 2 Ministry of Health and Family Welfare, Dhaka, Bangladesh; 3 Columbia University Irving Medical Center, NY, USA; 4 Resolve to Save Lives, New York, NY, USA; 5 University of California at San Francisco, San Francisco, California, USA

**Keywords:** hypertension control, WHO-HEARTS, Bangladesh, primary health care, electronic medical records, noncommunicable diseases

## Abstract

To improve hypertension control and prevent cardiovascular disease in Bangladesh, the National Heart Foundation of Bangladesh and Resolve to Save Lives collaborated with the Ministry of Health and Family Welfare to launch the Bangladesh Hypertension Control Initiative (BHCI). The BHCI implemented the WHO-HEARTS hypertension control package in four primary health care facilities (Upazila Health Complexes or UHCs) in 2018, then scaled up to 51 UHCs in 2021, 172 UHCs by 2024, and 320 UHCs by May 2026, covering about 54 million of 108 million adults (~43% coverage). By May 2026, the BHCI had enrolled 739,057 hypertensive patients and achieved 59% blood pressure (BP) control (<140/90 mmHg based on BP measured in the prior three months among all enrolled patients). The BHCI is a scalable model for hypertension control programs in other low- and middle-income countries. For sustained high-quality national scale-up, uninterrupted supply of medicines by the government and accurate information systems are crucial.

## Graphical abstract

**Figure:**
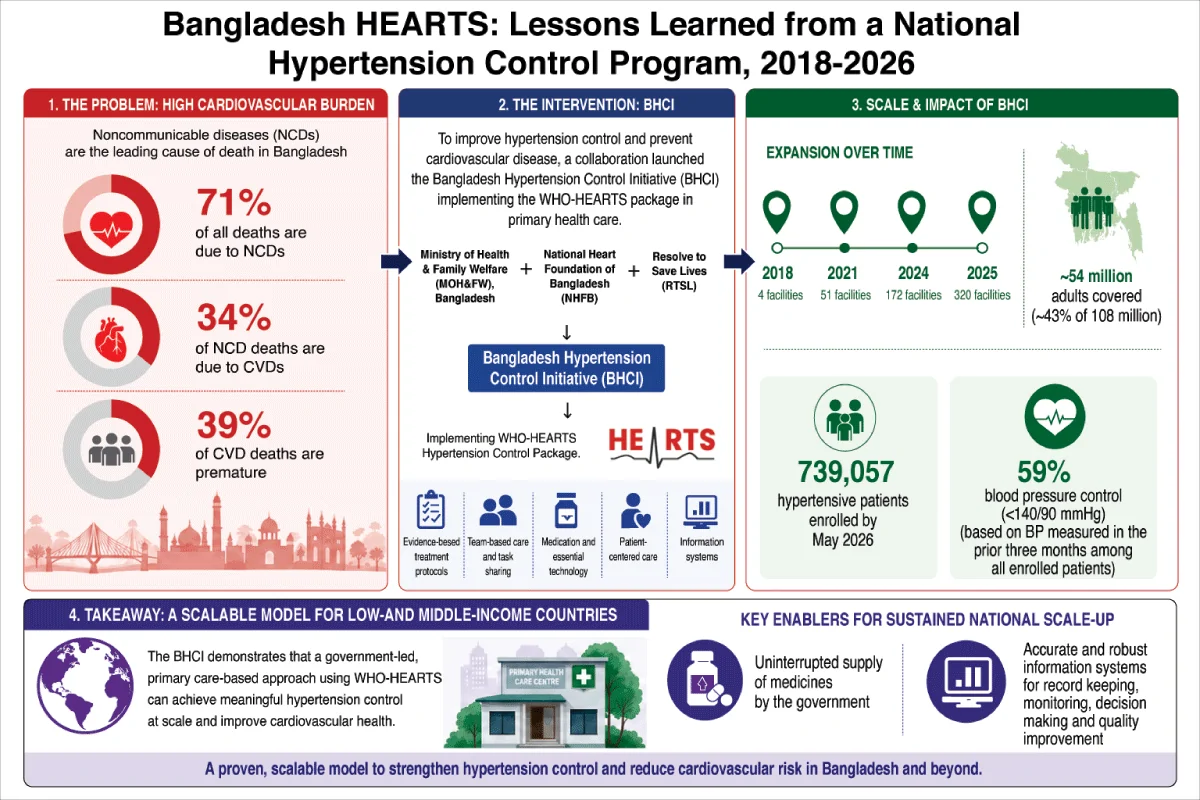


Bangladesh HEARTS: implementation, scale-up, outcomes, and key lessons (2018–2026).

## Introduction

Noncommunicable diseases (NCDs) account for 71% of all deaths in Bangladesh, with cardiovascular disease (CVD) accounting for 34% of NCD deaths (1). Uncontrolled hypertension is the leading preventable risk factor for CVD (2,3), with hypertension prevalence increasing from about 20% in 2018 to 25% in 2022 (4,5). Among the estimated 18.87 million adults with hypertension in 2018, 51% were unaware of their raised blood pressure (BP), 21% were on treatment, and 14% had their BP controlled (<140/90 mmHg) (4). This challenge reflects a global trend: in 2024, hypertension affected about 1.4 billion adults worldwide—double the number in 1990—but only 320 million (23%) had their blood pressure under control. While Canada, Costa Rica, Iceland, and the Republic of Korea have achieved population BP control rates above 50%, 99 nations remain below 20%. Bangladesh’s control rate of 16% underscores both the national and regional burden of hypertension and the urgent need for effective prevention and management strategies (6).

In 2018, the Ministry of Health and Family Welfare (MoH&FW) adopted the Multisectoral Action Plan for the Prevention and Control of Noncommunicable Diseases (MSAP), aiming to reduce the disability and premature mortality associated with NCDs. Its core objectives include scaling up multisectoral responses to NCDs, enhancing community capacity for health literacy and prevention, strengthening primary health care (PHC) for early detection and treatment, and establishing robust surveillance and monitoring systems to inform evidence-based policy and programming. The MSAP’s proposed targets include a 25% relative reduction in the prevalence of raised BP and a 25% relative reduction in overall mortality from NCDs (7,8).

To achieve these national objectives, Bangladesh has prioritized strengthening its PHC system, which is organized into a tiered network of facilities serving rural populations. The Upazila Health Complexes (UHCs) and Union Sub-Centers (USCs) operate under the Directorate General of Health Services (DGHS) and are staffed by physicians, nurses, and Sub-Assistant Community Medical Officers (SACMOs). Community Clinics (CCs), managed by the Community Clinic Health Support Trust (CCHST), are operated by a single Community Health Care Provider (CHCP) and offer frontline services in rural areas (9).

To ensure integrated NCD management, the Non-Communicable Disease Control (NCDC) program of the DGHS established dedicated ‘NCD Corners’ as one-stop service points for comprehensive NCD care at UHCs. At the community level, community clinics serve as frontline platforms for early screening and preventive interventions, focusing on rural and underserved populations (8).

To address gaps in hypertension detection and treatment and prevention and control of CVDs, the NCDC, in collaboration with the National Heart Foundation of Bangladesh (NHFB) and Resolve to Save Lives (RTSL), established the Bangladesh Hypertension Control Initiative (BHCI) in 2018 as a national adaptation of the WHO-HEARTS technical package to strengthen detection, treatment, and control of hypertension in PHC (10). This paper describes the components, implementation strategies, evolution, results, and lessons learned in implementing the BHCI (Figure 1).

**Figure 1:**
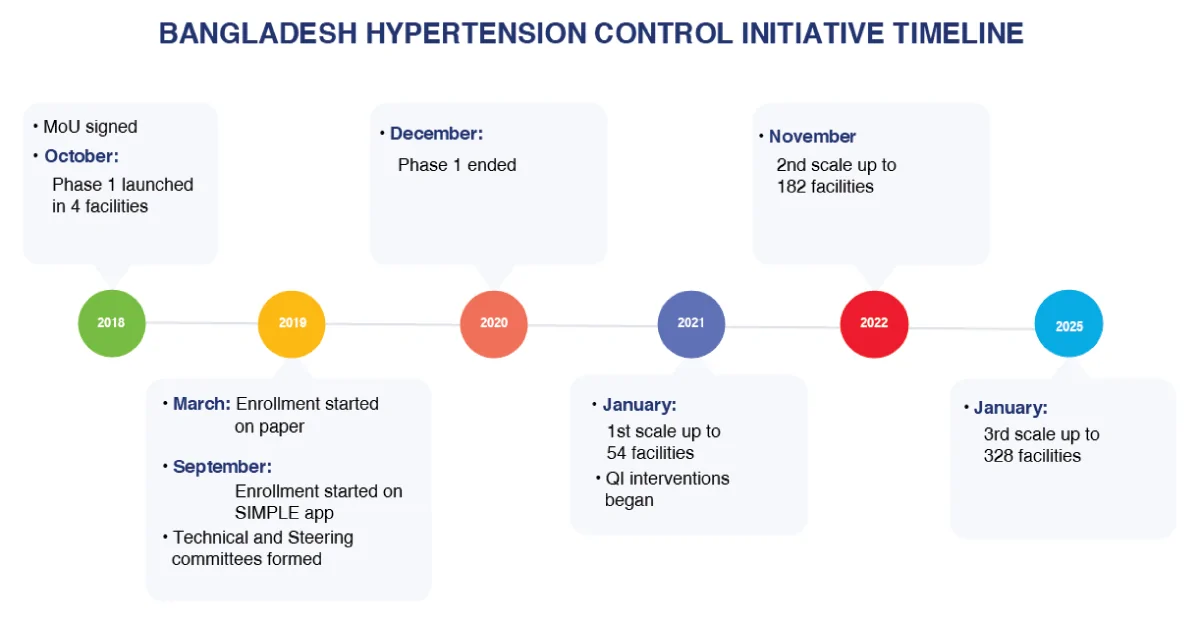
Timeline of key activity highlights in the Bangladesh Hypertension Control Initiative.

## Bangladesh Hypertension Control Initiative: Components and Implementation Strategies

### BHCI components

The BHCI is a country-adapted version of the WHO-HEARTS technical package (10), a suite of effective and scalable interventions for hypertension control in PHC settings. HEARTS offers an alternative to the acute, episodic orientation of health systems in low- and middle-income countries, widely recognized as a barrier to effective NCD care. Instead, it promotes an integrated approach that emphasizes prevention, diagnosis, and lifelong treatment (11). The BHCI operationalized HEARTS into the five pillars summarized in Table 1.

**Table 1:** Components of the Bangladesh Hypertension Control Initiative.


**Evidence-based treatment protocols**	Adoption of a simple, linear, drug- and dose-specific protocol for use in primary care settings through multiple national-level stakeholder meetings. Protocol uses three of the most effective and readily available antihypertensives.

**Medication and equipment supply**	System to dispense 30-day supply of antihypertensive medicines free of charge to patients. Use of validated digital BP measuring devices.

**Team-based care**	Hypertension diagnosis and management by a multidisciplinary team at the NCD Corner of each UHC. Clear two-way referral pathway between the UHC and community clinics.

**Patient-centered services**	Universal facility-based BP measurement. Patient education and lifestyle modification counseling. Service decentralization and referral of stably controlled patients to community clinics for drug refill.

**Information systems**	A user-centered smartphone health information system to collect and manage a limited set of patient data over time. Rapid feedback loops and performance monitoring using program data to improve care delivery.


#### Evidence-based treatment protocols

Adoption of a simple, linear, and drug- and dose-specific hypertension treatment protocol containing three of the most effective antihypertensive medicines readily available in the country is a key measure. The protocol (Figure 2) begins with amlodipine, a calcium channel blocker selected for its affordability, efficacy, and low risk of electrolyte imbalance, which eliminates the need for laboratory monitoring. If patient BP remains uncontrolled on amlodipine 5 mg alone, additional medicines are added, as per the protocol, at subsequent steps every four weeks until BP is controlled to <140/90 mmHg. Consistent with WHO treatment guidelines, the BP control target for patients with comorbidities such as diabetes, ischemic heart disease, stroke, chronic kidney disease, or high CVD risk is <130/80 mmHg. If the target BP is not achieved by step three and after confirming treatment adherence, patients are referred to specialist management.

**Figure 2:**
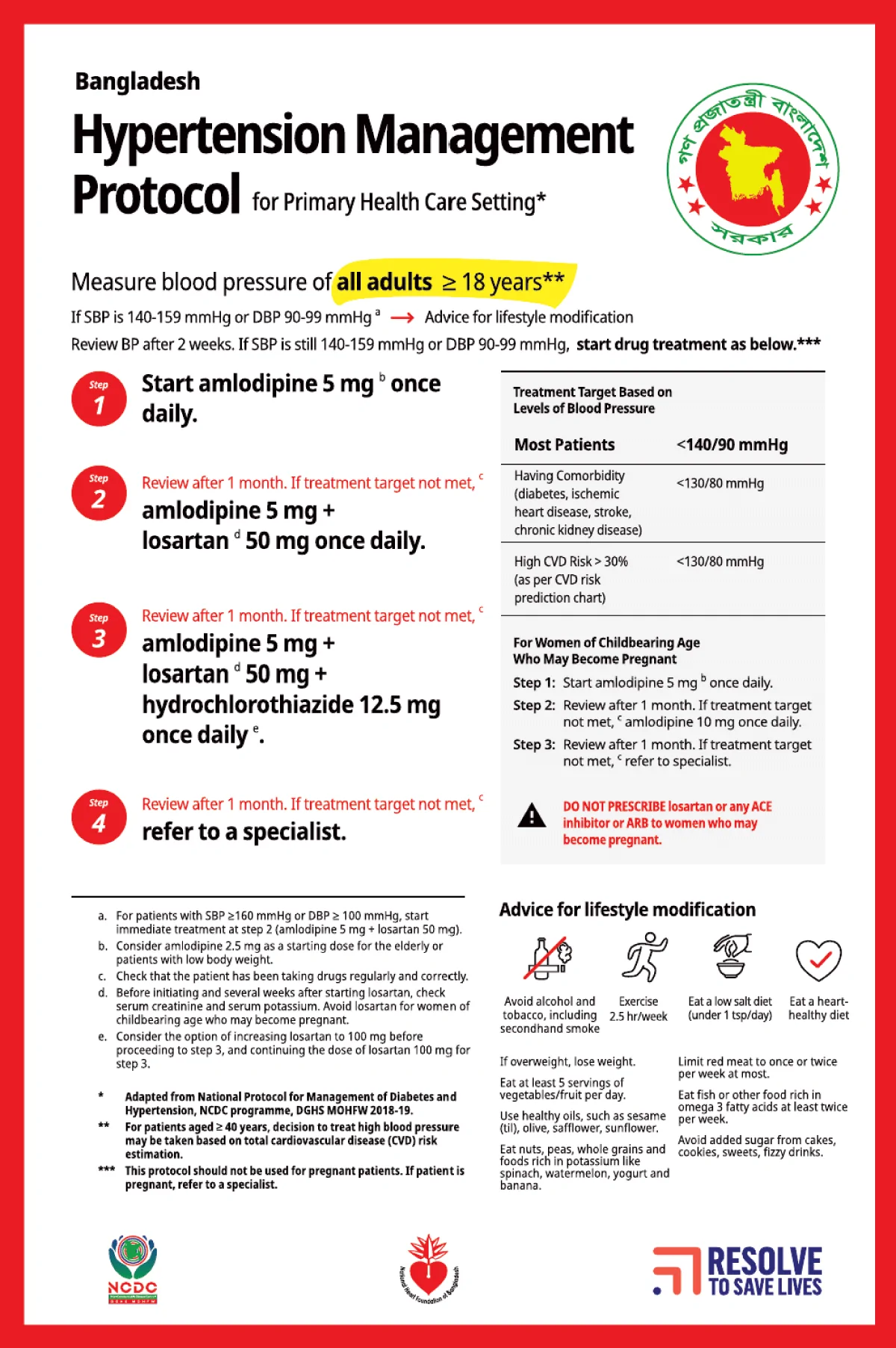
Hypertension management protocol for primary health care settings in Bangladesh.

#### Team-based care and task sharing

A multidisciplinary team of appropriately trained health care workers (HCWs) has strengthened and institutionalized hypertension diagnosis and management at the facilities. They work collaboratively at the NCD Corner under the supervision of a doctor. The team includes nonphysician members such as nurses, SACMOs, and pharmacists. Doctors are responsible for confirming diagnoses and initiating treatment in accordance with standardized treatment protocols. Nonphysician team members conduct patient screening, register new patients, document follow-up visits, refill medication, and provide counseling on medication adherence and lifestyle modifications. CHCPs also work in the community to increase screening, detection, referral, and follow-up of patients in selected CCs.

#### Medication and essential technology supply

A system has been established to dispense a 30-day supply of antihypertensive medications to all patients free of charge. Uninterrupted availability of medicine was ensured through strengthening the medicine forecasting and quantification system. All the NCD Corners were also equipped with validated digital BP measuring devices.

#### Patient-centered care

Service delivery elements have been incorporated to reduce barriers to treatment uptake, adherence, and retention. This includes universal BP measurement for all adults visiting the UHC, incorporating patient counseling and education on lifestyle modifications and medication adherence into routine clinic workflow, establishing systems for patient recall and follow-up to improve retention in care, decentralizing medication refills, and follow-up for stably controlled hypertensive patients at CCs to reduce barriers to access and alleviate congestion at the UHCs.

#### Information systems

A user-centered, smartphone-based health information system (the Simple mobile app) is being used to collect and manage a limited set of patient data longitudinally, enabling rapid feedback loops using program data and performance monitoring (12).

### BHCI implementation strategies

The BHCI employs a unified, scalable implementation strategy to enhance hypertension diagnosis and control within the public PHC system, aligning with the Government of Bangladesh’s MSAP for NCD control. This strategy integrates evidence-based interventions, facility-based screening, diagnosis, treatment, adherence support, and community engagement designed for iterative expansion across UHCs and CCs (summarized in Table 2).

**Table 2:** Phased BHCI implementation strategies.


IMPLEMENTATION STRATEGY	PHASE 1 OCTOBER 2018–DECEMBER 2020	PHASE 2 JANUARY 2021–OCTOBER 2022	PHASE 2 EXPANSION NOVEMBER 2022–DECEMBER 2024	PHASE 3 JANUARY 2025–MAY 2026

**Number of facilities (target)** ^a,b^	4	54	182	328

**Population coverage (adult)** ^a^	617,015	9,729,922	35,638,781	54,324,602

**Number of facilities (activated)** ^a,b^	4	51	172	320

**NCD Corner activation**	✓	✓	✓	✓

**Partnership and catalytic funding**	✓	✓	✓	✓

**Situation analysis**	✓	✓	✓	✓

**Development of hypertension treatment protocol for PHC**	✓			

**Facility assessment**	✓	✓	✓	✓

**Hypertension management training and capacity building**	✓	✓	✓	✓

**Patient flow and clinical decision streamlining**	✓	✓	✓	✓

**Systematic follow-up and reminder**	✓	✓	✓	✓

**Information system optimization**	✓	✓	✓	✓

**Implementation monitoring and supervision**	✓			

**Quality improvement**		✓	✓	✓

**Medicine refilling from CCs**		✓	✓	✓

**Community-based HEARTS**			✓	

**Improvement of medicine quantification and forecasting**	✓			

**Dissemination and documentation**	✓	✓		

**Stakeholder engagement and advocacy**	✓	✓	✓	✓


a – Population and facility coverage numbers in each phase are cumulative. b – Facilities were under renovation during the corresponding phases, but were activated later.

#### NCD Corner activation

To facilitate access to hypertension treatment, the government reactivated existing NCD Corners and made them the center of hypertension treatment service delivery at each UHC. The NCD Corners at each UHC are located on the ground floor close to the facility entrance for accessibility, have ample space to accommodate waiting patients, and adequate space to allow service delivery (9). The Government of Bangladesh renovated and equipped each NCD Corner with one arm-in heavy-duty digital BP device with a printer; two portable validated digital BP measuring devices; one aneroid BP machine; a weighing scale; a stadiometer; a measuring tape; chairs and desks; filing cabinets; and Information, Education, and Communication material display boards. A multidisciplinary team of appropriately trained HCWs collaborates at the NCD Corner under a doctor’s supervision to establish team-based care and task sharing. All steps are performed by appropriately trained nonphysician HCW team members, except for diagnosis and treatment initiation, which are performed only by a doctor.

#### Screening, diagnosis, and treatment

Opportunistic screening for raised blood pressure among all people aged 18 years or more visiting the center is carried out by nurses at NCD corners. The physician confirms the diagnosis and initiates treatment in accordance with the hypertension management protocol for PHC settings.

#### Record keeping and follow-up

Diagnosed patients are enrolled in the Simple app digital health information system and assigned a follow-up date one month later. Patients visit the NCD Corner monthly on the designated date for follow-up and medication refill.

#### Free medicine and longer refill duration

Patients receive one month of prescribed medicine, free of charge, provided by the government. This reduces the number of visits patients must make to the UHC. Previously, facilities dispensed only 1–2 weeks of medication at a time; however, following a directive from the DGHS, patients began receiving a full month’s supply with each prescription and during follow-up.

#### Counseling

Nurses provide patients with targeted counseling on key aspects of hypertension management, including adherence to prescribed medications, the importance of regular follow-up visits, and practical healthy lifestyle advice.

#### Partnership and catalytic funding

The Government of Bangladesh recognized that it would need an effective model for hypertension management at the PHC level to treat the millions of people living with hypertension and achieve hypertension control targets. To accomplish this, the government partnered with the NHFB and RTSL to design, fund, and manage the BHCI program. The NCDC program provides strategic guidance and overall coordination, while the NHFB provides comprehensive implementation strategies, including policy engagement, monitoring and evaluation frameworks, supervisory mechanisms, and continuous quality improvement (QI) approaches. In addition, the NHFB also serves as the institutional hub for program management infrastructure. RTSL provides hypertension management expertise, access to the Simple mobile app digital information system, and technical guidance on program evaluation and QI activities.

The Government of Bangladesh provides facility infrastructure, BP monitors, antihypertensive medicines free of cost to patients, and HCWs’ salaries. RTSL’s supplemental catalytic funding provided a start-up supply of validated digital BP monitors, salaries of program management and supervisory staff, and funding for HCW training.

#### Situation analysis and protocol development

A comprehensive analysis of existing hypertension services, including epidemiology, treatment guidelines, protocols, and training materials, was conducted before implementation. An expert committee initially developed the national hypertension treatment protocol for PHC, which was subsequently approved by the Technical Committee to standardize care delivery across facilities (Figure 2).

#### Facility assessment

NCD service preparedness of health facilities was evaluated using an adapted WHO Service Availability and Readiness Assessment (SARA) questionnaire focusing on infrastructure, equipment, and staffing (13). Facility assessments conducted in 2021, during phase 2 at the sub-district level and including facilities from phase 1, reported that only 17.6% of facilities met the criteria to be considered service ready. Also, none of the facilities had a dedicated room for NCD management (14). Following the implementation, each facility now maintains a dedicated ‘NCD Corner’ for NCD management.

#### Hypertension management training and capacity building

To promote team-based care and ensure that all team members were adequately skilled in hypertension treatment (15), the program trained all relevant cadres of HCWs at the UHCs and CCs, including doctors, nurses, SACMOs, health inspectors, assistant health inspectors, storekeepers, statisticians, and community health care providers. These comprehensive training sessions covered the NCD and hypertension burden, hypertension diagnosis including appropriate BP measurement techniques, hypertension treatment according to the PHC protocol, patient follow-up and recall strategies, and establishing NCD Corner workflows to optimize BP measurement, patient care documentation processes, and data use for program improvement. Refresher training is held to account for staff attrition and knowledge loss over time.

#### Patient flow and clinical decision streamlining

To increase hypertension detection among patients visiting UHCs, all adult patients (18 years and older) are first directed to the NCD Corner for BP screening regardless of the reason for their visit. The program also adopted clear diagnostic criteria to facilitate early diagnosis and treatment initiation. If the initial BP is <140/90 mmHg and patients are not receiving antihypertensive medicine, they are regarded as normotensive. If, at initial screening, systolic blood pressure (SBP) is 140–159 mmHg and/or diastolic blood pressure (DBP) is 90–99 mmHg, patients are advised to return for a follow-up visit after two weeks for a repeat BP measurement. If the repeat BP is still above 140/90 mmHg, a diagnosis of hypertension is confirmed, and patients are enrolled in the Simple app. If, at the initial screening, SBP is ≥160 mmHg and/or DBP ≥100 mmHg, a repeat BP measurement is taken the same day to confirm diagnosis and start treatment immediately. Newly diagnosed hypertension patients are provided with one month of prescribed medicines free of cost and advised to follow up at the facility one month later. NCD Corner staff also provide advice for healthy lifestyle interventions to control high BP (Figure 3).

**Figure 3:**
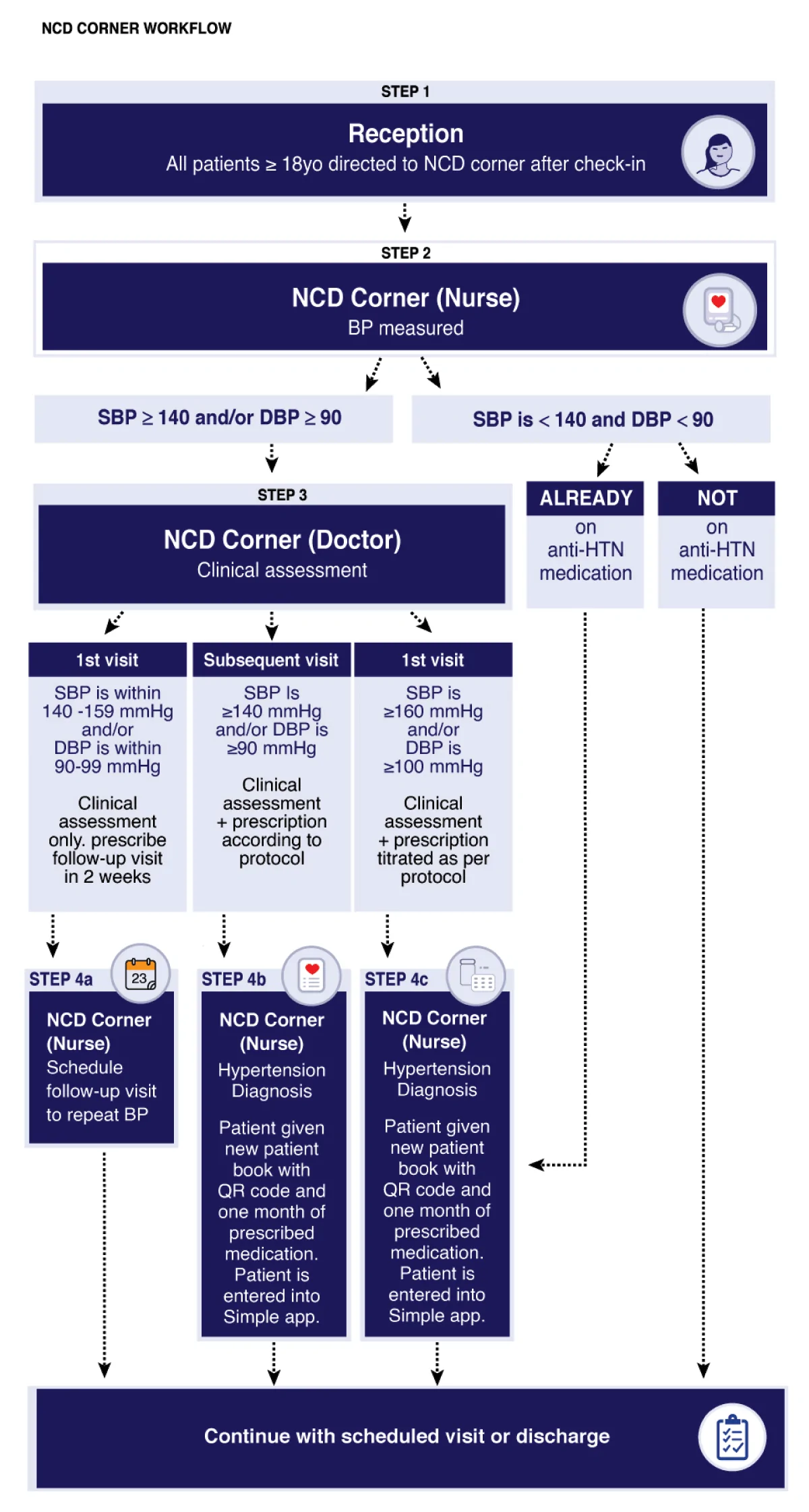
NCD Corner workflow for hypertension screening, diagnosis, treatment, and follow-up at the NCD Corner.

#### Systematic follow-up and reminder

To strengthen patient retention and improve adherence, the BHCI employs a dual strategy of digital and human outreach. Automated reminder messages are sent to patients who miss scheduled visits, resulting in a higher follow-up visit attendance rate and reducing delays in care (16). Complementing this system, BHCI operates a dedicated call center that conducts personalized phone outreach to patients with overdue or missed visits.

#### Information systems optimization

To ensure continuity of care, data use, and accountability for patient outcomes, the BHCI utilizes the Simple mobile app, an open-source, smartphone-based hypertension management information system also used in Ethiopia and Sri Lanka (12,17). This digital system swiftly records patient data in about 83 seconds for new patient enrollment and less than 20 seconds for follow-up visits (18), which reduces HCW data entry time by approximately 20 minutes per day compared to the prior paper-based system. Simple’s offline-first feature allows functionality in facilities without internet connectivity. The software has two components: 1) an Android mobile phone app for health care providers to record patient BPs, prescriptions, and follow-up visit dates at every visit, which provides a longitudinal patient record and the capacity to monitor an individual patient’s response to treatment (Figure 4, Panel A); and 2) a web-based dashboard for health system managers at the facility, district, and national levels to monitor program performance trends and plan improvement efforts (Figure 4, Panel B).

**Figure 4:**
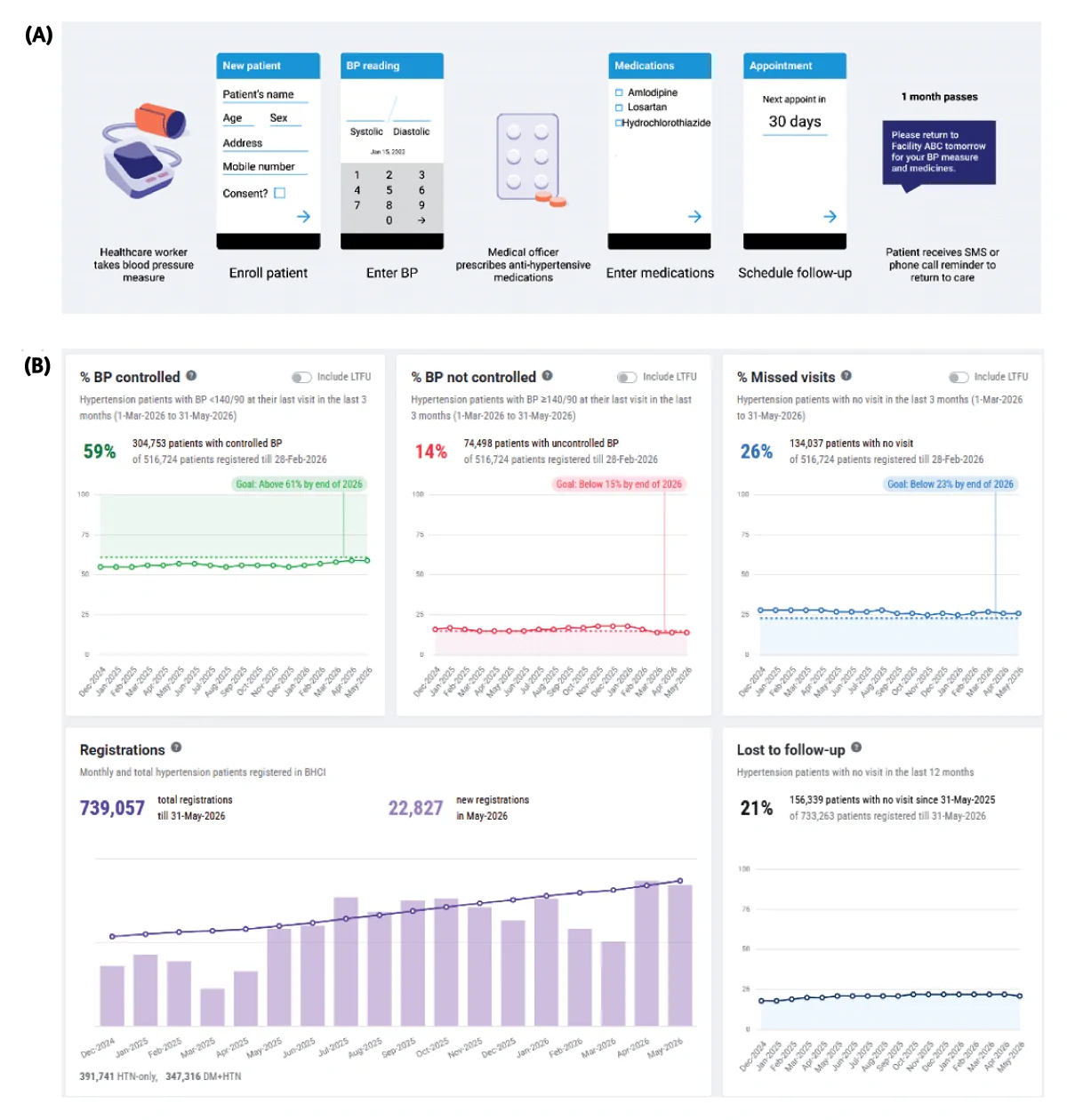
**(A)** Screenshots illustrating the Simple mobile app smartphone user interface; **(B)** Program-level dashboard for monitoring performance.

The BHCI provided Android devices and monthly data packs for all facilities within the program area, enabling them to enroll patients using the Simple app at NCD Corners. At enrollment, patients provide their National ID card number and phone number, which are recorded on the Simple app, and each patient receives a paper hypertension program card (BP passport) with a QR code sticker. At return visits, HCWs scan the QR code with the Simple app to instantly retrieve patient records. The Simple app also generates a list of patients overdue for scheduled follow-up appointments and provides features for conducting secure telephone calls and sending automated SMS reminder messages. Facility and program managers utilize the Simple dashboard to manage resources, monitor patient demand, observe trends in key indicators, identify gaps in care, and track hypertension control rates across the region.

To promote focus on BP control and avoid overburdening frontline HCWs with reporting requirements, the BHCI chose to focus only on the most critical indicators needed to monitor program performance and patient outcomes. These include the number of people living with hypertension enrolled, the proportion with BP controlled, the proportion with BP not controlled, the proportion with missed visits, and patient days of medicines available per facility (definitions included in Supplementary Table S1).

#### Implementation monitoring and supervision

Implementation monitoring in the BHCI involves periodic, data-driven tracking of activities and outputs against planned targets to inform timely corrective actions. It uses tools such as dashboards, indicator matrices, and routine reports as demonstrated by the HEARTS360 framework, which translates large patient datasets into actionable insights (19). Insights derived from supervisory visit documentation and aggregated data also help identify implementation gaps and challenges. Government officials oversee the program through four committees: two policy-level committees for governance and alignment, and two operational committees for coordination and service delivery.

The BHCI supervision framework operates through a multi-tiered structure. At the frontline, Field Monitoring Assistants (FMAs) provide direct oversight and guidance to NCD Corner teams, aiming to enhance service delivery, ensure adherence to clinical standards, and build capacity through face-to-face engagement. At the intermediate level, Divisional Program Officers (DPOs) address administrative challenges, including drug stock monitoring and communicating with the call center. At the same time, Surveillance Medical Officers (SMOs) supervise FMAs, arrange feedback sessions and on-the-job training for NCD Corner team members, and provide technical support using structured tools such as the Simple dashboard and checklists to identify and address implementation gaps.

These officers maintain regular collaboration with facility, district, and divisional managers to resolve operational bottlenecks. At the national level, Program Directors oversee strategic supervision, engage with policymakers and stakeholders, and guide programmatic decisions, while ensuring capacity development of program staff. The BHCI’s supervision approach is inherently interactive, emphasizing real-time observation, contextual problem solving, and translating data monitoring into actionable improvements across the health system.

#### Quality Improvement

To further drive data use to improve patient outcomes and program performance, the BHCI introduced a systematic QI program embedded within supervisory structures at national, sub-national, and facility levels. DPOs and SMOs work with facility staff to review data on the Simple app to identify performance gaps, conduct root-cause analyses, plan and implement small tests of changes, and scale up successful interventions across all participating facilities. Program leadership meets monthly to review program performance and provide technical support and QI mentorship to local teams. Divisional and District Coordination Committees are responsible for providing QI oversight at PHC facilities. Monthly facility QI meetings are used to review data and develop iterative action plans. Outcomes of BHCI QI initiatives are provided in Table 3.

**Table 3:** BHCI quality improvement interventions and outcomes, July 2020–May 2026.


INDICATORS	COUNTERMEASURES	ACTIVITIES	MEASURING TOOLS	OUTCOME

**Control rate**	Training for Health Service Providers (HSPs)	Training at District/UHC/CC level	Number of HSPs trained	BP control rate increased from 37% in July 2020 to 59% in April 2026

	Data sharing with HSP	QI meetings, data wall display	Number of QI meetings, number of facilities using data wall	

	Patient counseling by nurses	Advocacy with the nurses to counsel the patients accordingly	Observation	

	Reducing patient waiting time	Digital token box, NCD volunteer	Counting patient time starting from entry to exit at the facility	

	Therapeutic inertia/drug escalation	Simple app clinical decision support (CDS) alert, red sticker	Drug titration rate for patient visits with uncontrolled BP	

	Drug redistribution	From surplus facilities to stockout facilities	BHCI monthly update	

	Drug prediction tools development	Ongoing	Ongoing	

**Missed visits**	Telephone calls to overdue patients	Field Monitoring Assistants (FMAs) called overdue patients through Simple app, establishment of NHFB centralized call center	Proportion of overdue patients called and returned to care each month	Missed visits reduced from 53% in July 2020 to 26% by May 2026

	Text messaging to overdue patients	Text sent to the patient’s registered phone number from Simple app	Number of text messages sent to the patients	

	Decentralizing care to CCs	CHCP training, patient assignment by nurses, patient assignment by FMAs	Number of eligible patients assigned to CCs, follow-up and control rate at CCs	

	Community engagement	Advocacy meeting with Union Chairs, Members, CG, and CSG		

	Universal screening	Instruction from ticket counter referring patients to NCD Corner	Number of patients registered through Simple app	

**Patient registration**	Community awareness program	Advocacy meeting at Union parishad with community leaders	Number of meetings held in program areas	Total registrations increased from 12,749 in July 2020 to 739,057 (4% community coverage) by June 2026

	Community awareness program CHCP training	Advocacy meeting with CG/CSG	Number of meetings held in program areas	

		CC referral	Number of referred patients at NCD Corner	

	Media coverage	News coverage in electronic and print media	BHCI monthly program update	

	Day celebration	Health campaign, MMM	Data collection	


#### Medicine refills at community clinics

The BHCI piloted a decentralized antihypertensive medication refilling model across 107 CCs affiliated with five UHCs in the Sylhet district. This intervention targeted patients with controlled BP, enabling them to access medication refills directly from CCs rather than traveling to UHCs. No formal diagnoses or prescriptions were issued at CCs, which operate without on-site medical officers.

The pilot revealed that proximity to home and reduced waiting times made CCs more convenient and accessible for patients, contributing to improved service uptake. Monitoring data from the pilot sites indicated a BP control rate of 68% and a missed visit rate of 21%, compared to the district control rate of 57% and missed visit of 31%, underscoring the potential of community-based refilling models to strengthen continuity of care and optimize hypertension management.

#### Community-based HEARTS pilot

While the Bangladesh HEARTS trial focused on implementation at the UHC level, further evidence was needed of its effectiveness at the CC level. To address the challenges of serving a large number of hypertensive patients with a comparative shortage of trained physicians and nurses, and to reduce the burden of long-distance travel for monthly UHC visits for patients with controlled BP, a decentralized, team-based hypertension care model was piloted in CCs beginning in 2023. In this model, nonphysician staff screened and managed patients using HEARTS protocols under remote supervision from UHC Medical Officers via tele-consultancy. Conducted in four CCs under two UHCs in Jamalpur, the pilot screened 4,220 adults and enrolled 220 newly diagnosed, uncomplicated hypertension patients out of 244 eligible patients. The findings from this pilot are soon to be published.

#### Improvement of medicine quantification and forecasting

The Essential Drugs Company Limited (EDCL), operated by the Government of Bangladesh, supplies antihypertensive medicines based on demand estimates submitted by facility managers to the NCDC program. BHCI supervisory staff prepare the estimates using data that include patient volume and the proportion of patients on different protocol steps. This data-driven, prevalence-based process replaced the previous consumption-based quantification approach to help prevent stockouts and ensure consistent hypertension care at the PHC level.

#### Dissemination and documentation

Good practices, outcomes, and lessons learned are documented regularly and disseminated through national and district-level meetings with MoH&FW leaders, members of parliament, and health officials. Insights are also shared with policymakers and stakeholders to inform the scale-up and policy integration process.

#### Stakeholder engagement and advocacy

The BHCI has prioritized strategic collaboration across multiple governance levels to ensure adequate oversight and sustained stakeholder engagement. Facility visits and regular program progress updates are utilized to engage members of the Steering and Technical Committees, officials from the DGHS and MoH&FW, regulatory bodies, and local leaders, which contributes to strengthening institutional support and accountability.

To boost engagement, community programs were organized involving community groups, community support groups, and CHCPs. Participants received an introduction to NCD care at the UHCs and were encouraged to promote regular BP measurement, adopt healthy lifestyles, and use the NCD Corner services. Over 5,000 CCs benefited, resulting in increased BP screening, stronger referral systems, and expanded community awareness efforts.

## Evaluation of the BHCI

### Effectiveness of the HEARTS package compared with usual care

To assess the effectiveness of the BHCI WHO-HEARTS hypertension control program, a matched-pair cluster quasi-experimental trial was conducted in 2021–2022 (20). A total of 3,935 patients with hypertension were enrolled in the study, with 1,950 in the intervention group and 1,985 in the usual care group. Compared with usual care, the intervention significantly lowered systolic BP (–23.7 mm Hg vs. –20.0 mm Hg; net difference –3.7 mm Hg [95% CI: –5.1 to –2.2]) and diastolic BP (–10.2 mm Hg vs. –8.3 mm Hg; net difference –1.9 mm Hg [95% CI: –2.7 to –1.1]) and improved hypertension control (62.0% vs. 49.7%, net difference 12.3% [95% CI: 9.0 to 16.8]). The rate of missed clinic visits was lower in the intervention group (8.8% vs. 39.3%, p < 0.001).

The Bangladesh HEARTS trial showed that the BHCI significantly reduced systolic and diastolic BP, improved BP control, and enhanced retention in care. A prior costing study found that implementing the HEARTS package lowered patient out-of-pocket expenses and improved medication adherence (21).

### Bangladesh HEARTS costs and budget impact

Effectiveness and cost data from the Bangladesh HEARTS program were translated into national projections to inform scale-up decisions. Using facility-based hypertension control rates, a web-based model estimated that nationwide implementation over a 10-year period would require approximately US$599 million in cumulative public-sector expenditure by 2030. During this period, improved primary care hypertension control is estimated to avert 9,400 CVD deaths while raising the national hypertension control rate from 13% to 33%. Scenario analyses indicate that a 50% reduction in antihypertensive medication prices would decrease costs by 42.6%, and the elimination of routine laboratory monitoring would decrease costs by 14%. Both strategies, in combination with enhanced task-sharing (expanded roles for nurses and community health workers), would decrease costs by up to 56.9%. Team-based care alone would exert a negligible budgetary impact (22).

## Results

### Hypertension indicators

*Phase 1: October 2018–December 2020*. At the end of phase 1, the total number of hypertensive patients was 18,217, the control rate was 49%, and the missed visit rate was 43% (Figure 5).

**Figure 5:**
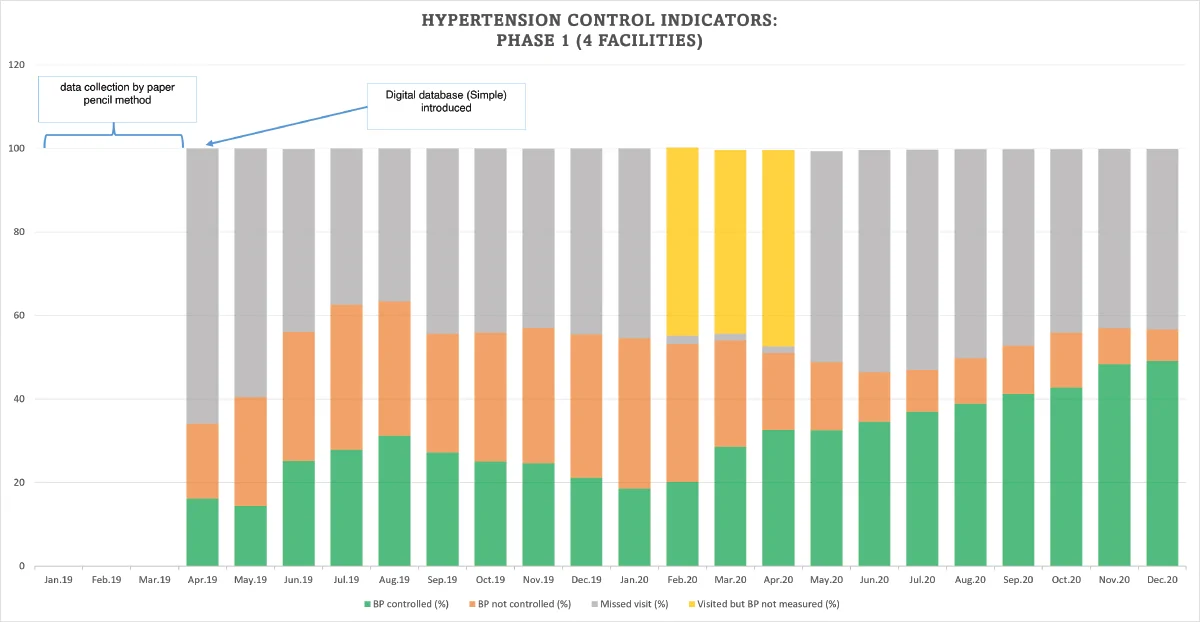
BHCI hypertension control performance indicator status at the end of phase 1. * Before April 2019, patient records were maintained using paper-based records. In April 2019, a digital database (Simple) was introduced, which became the cornerstone for monitoring program indicators. From February 2020 to April 2020, BP could not be measured for all follow-up patients due to government-implemented COVID-related lockdowns. Patients visited health care facilities and refilled medicine without measuring their BP.

*First scale up – Phase 2: January 2021–October 2022*. At the end of phase 2, the total number of hypertensive patients was 109,282, the control rate was 60%, and the missed visit rate was 26% (Figure 6).

**Figure 6:**
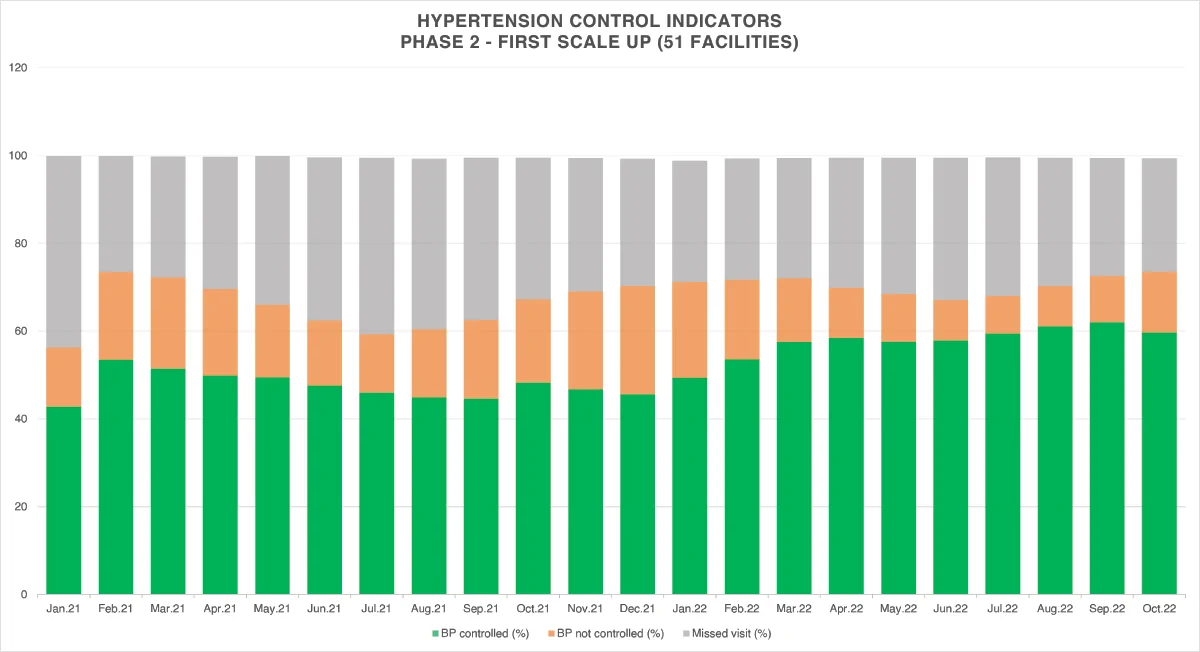
BHCI hypertension control indicator status at the end of phase 2.

*Second scale-up – Phase 2 Expansion: November 2022–December 2024*. In December 2024, the total number of hypertensive patients was 455,558, the control rate was 55%, and the missed visit rate was 28% (Figure 7).

**Figure 7:**
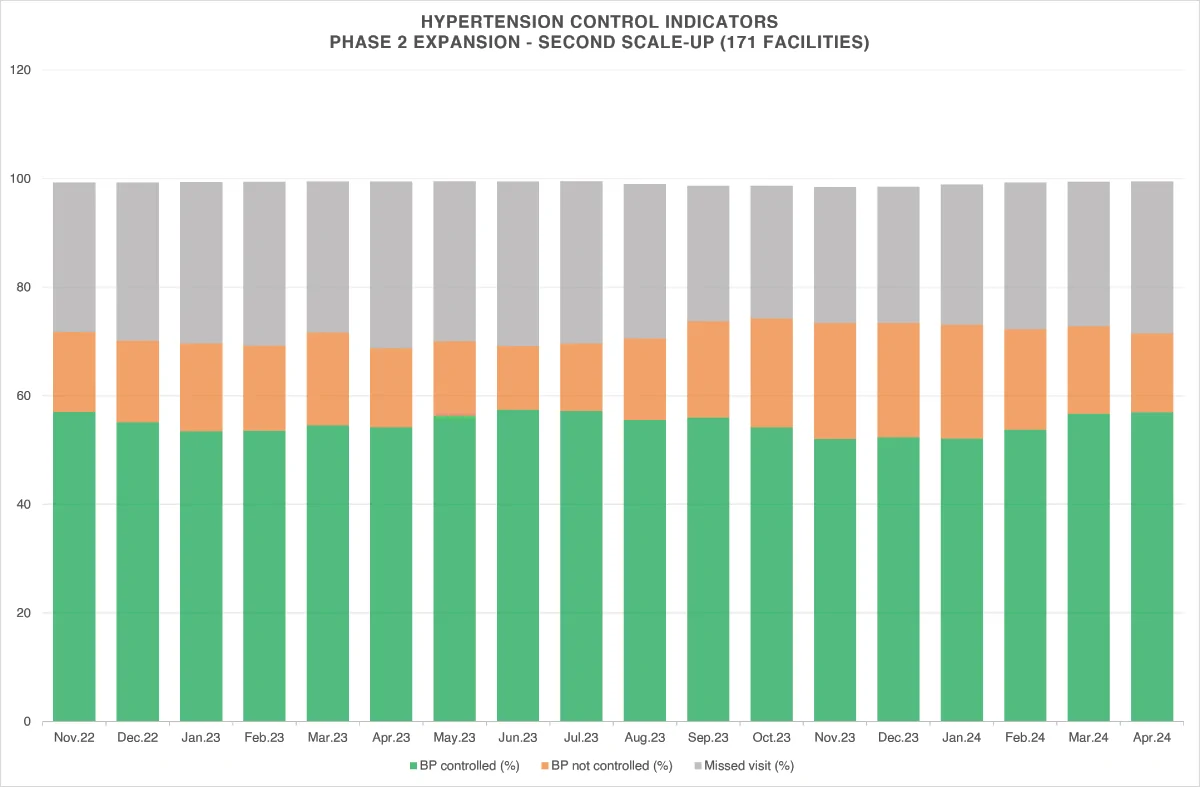
BHCI hypertension control indicator status through December 2024.

*Third scale-up – Phase 3: January 2025–May 2026*. In May 2026, the total number of hypertensive patients was 739,057, the control rate was 59%, and the missed visit rate was 26% (Figure 8).

**Figure 8:**
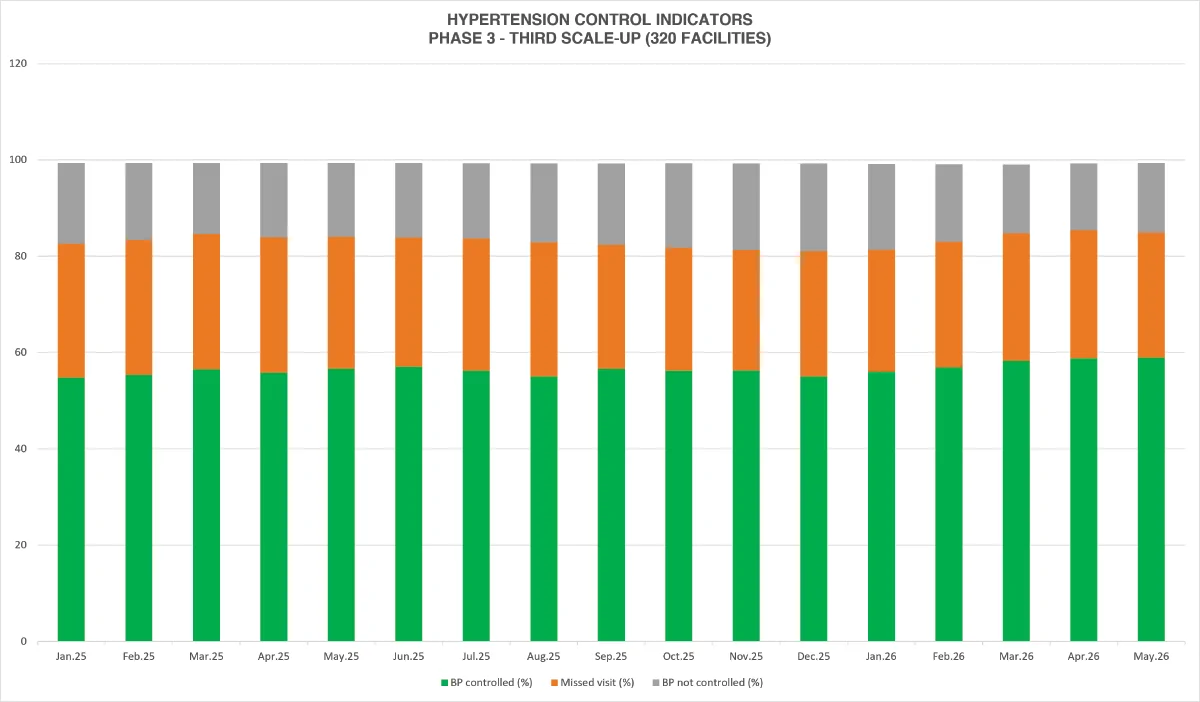
BHCI hypertension control indicator status through May 2026.

The COVID-19 pandemic disrupted the regular activities of both the BHCI and the Bangladeshi health care system, as well as patient behaviors, as seen in indicator trends. Apart from that, the program was able to maintain the BP control rate at around 50% throughout the program, even with the scale-up and increasing number of follow-up patients (Figure 9).

**Figure 9:**
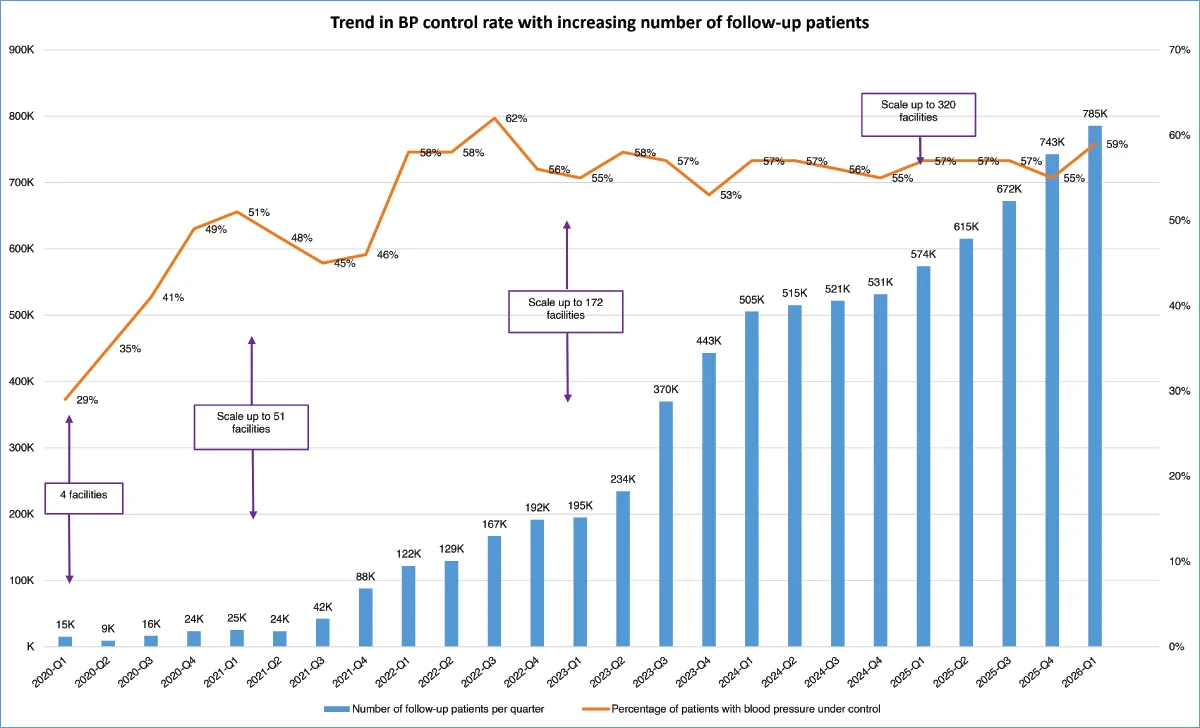
Trend in BP control rate with increasing number of patients being treated.

### Quality improvement outcomes

The BHCI QI approach developed and tested interventions to improve BP control, increase health care worker adherence to treatment protocols, reduce treatment inertia and loss to follow-up, enhance BP measurement and opportunistic screening, optimize NCD Corner activities and patient flow, and decentralize care to community clinics closer to patients’ homes. As a result, from the launch of QI in July 2020 through May 2026, BP control improved from 37% to 59%, missed visits declined from 53% to 26%, and patient registrations rose dramatically from 12,749 to 739,057.

Improving retention in care has been a central focus of the BHCI’s QI efforts. When the program began, 53% of patients had missed follow-up visits. Following introduction of patient call systems in July 2020, missed visits dropped to 26% within six months and have since remained below 30%. To enhance follow-up and visit attendance, the BHCI introduced automated SMS appointment reminders in 2021. A randomized A/B test revealed a 5.8% increase in attendance among overdue patients who received reminders (26.5% vs. 20.7%, p < 0.001) (16).

## The BHCI 2018–2026: Lessons Learned

### Importance of a favorable policy environment and strong government leadership

Bangladesh is a lower middle-income country amid an epidemiologic transition from infectious diseases to NCDs (23). The country is struggling with a HCW shortage, characterized by an imbalance in distribution that favors urban areas (24,25,26). The health system was also previously focused on maternal and child health as well as communicable diseases (27). Despite these challenges, the Government of Bangladesh is committed to prioritizing multiple NCD control initiatives, which emphasize NCD control as a key governmental agenda item (7).

Sustained collaboration with government stakeholders—from frontline HCWs to senior administrators—was pivotal to the BHCI’s progress. Familiarity with program operational dynamics fostered ownership and appreciation for its impact on hypertension control. Regular advocacy efforts reinforced the strategic importance of NCD management, demonstrating that strong policy alignment and institutional support are essential to achieve scalable health outcomes.

### Simple hypertension treatment protocol

The HEARTS initiative’s core innovation—simple treatment protocols—simplifies hypertension management training, supports a transition to team-based care, and leads to more affordable medicines through economies of scale. In Bangladesh, initial induction followed by refresher training enhances provider adherence to the national protocol, ensuring appropriate drug escalation or titration. Unpublished BHCI data from 2024 show high protocol adherence by HCW teams: 99% of patients receive protocol-based medications, with 76% prescribed amlodipine, 58% losartan, and 10% hydrochlorothiazide.

### Hypertension screening

Hypertension screening currently reaches only about 8% of adults, and facilities are already overcrowded (28). Universal opportunistic screening is efficient and low-cost, but it excludes those who rarely attend UHCs or CCs, contributing to persistently low rates of diagnosis and awareness. Repeat screening of non-hypertensive patients adds inefficiencies, while frequent staff transfers disrupt continuity, as newly posted providers often lack familiarity with HEARTS protocols and digital BP devices. With increased uptake, the challenge becomes not only the completeness of screening conducted across facilities, but also which facilities remain uncovered. Addressing this requires decentralization of services and multi-month prescribing, so that once patients are diagnosed and initiated on treatment, they can be managed closer to home with fewer visits, reducing pressure on overcrowded centers and improving long-term control.

### Hypertension medicine availability

A reliable supply of high-quality and affordable protocol medicines is essential for a successful HEARTS hypertension control program. The Government of Bangladesh provides antihypertensive medicines, among other drugs, free of charge to patients. However, the supply is at times not demand-based, as facilities lack accurate estimates of antihypertensive medication requirements for a growing hypertension control program.

Early in the BHCI program, facilities frequently experienced stockouts. This undermined patient trust in the reliability of medicine supplies, resulting in increased missed visits and a decline in control rates. Facilities were unable to obtain sufficient medicine supplies because they were required to procure medicines from the government-owned pharmaceutical firm, which continues to struggle with capacity expansion to meet growing program demands. To implement a sustainable hypertension control program, it is crucial to allocate a dedicated budget for medicines, develop an accurate morbidity-based medicine requirement forecasting tool, and operate an efficient supply chain. When these medication supply reforms are fully implemented and drug supplies are reliable, program costs will be better controlled.

### Retention in care

Consistent adherence to treatment and regular follow-up is essential for hypertension control, yet early BHCI data showed that over half of enrolled patients failed to return for follow-up. The QI initiative identified key barriers to retention and implemented targeted solutions: phone calls for missed visit patients, streamlined care routines to achieve BP control with fewer visits, and community advocacy to raise awareness about adherence, which resulted in significant improvements in patient retention (Table 3).

### Information system

There are at least 100 patient visits to NCD Corners every day during the morning shift at each of the BHCI-covered UHCs. For example, 785,000 patients visited NCD Corners within the program area in January–March 2026. In 2018, facilities relied on paper-based health records; however, this system proved inadequate for an extensive, national-scale program, as it not only wasted HCW and patient time but also hindered timely and accurate monitoring of treatment outcomes. Using a fast, user-centered digital information system, such as the Simple mobile app, has been vital to the BHCI, as it provides rapid feedback and focuses on key indicators to deliver quick program updates that drive QI efforts. Program managers and health managers can quickly identify areas for improvement and provide targeted interventions. Currently, the Simple database is being linked to the national DHIS2 database, which collects routine health data on all major health conditions from government health facilities across Bangladesh.

## Conclusions: The Future of HEARTS in Bangladesh

From 2018 to 2026, the BHCI developed, tested, and scaled up a standard WHO-HEARTS package to strengthen hypertension diagnosis, treatment, and follow-up in PHC facilities of rural Bangladesh. The Government of Bangladesh considers the BHCI HEARTS hypertension control program a model approach for hypertension management, suitable for implementation at all PHC facilities in Bangladesh. The evidence reviewed demonstrates that the BHCI has succeeded in advancing the goal of providing a reliable model for controlling hypertension and other NCDs, which will guide the nation’s expansion of NCD services. For sustained high-quality national scale-up, uninterrupted supply of medicines by the government is crucial. This project could also serve as an excellent model for implementing the HEARTS package in other countries with similar socioeconomic and health care contexts.

## Additional Files

The additional files for this article can be found as follows:

10.5334/gh.1595.s1 Supplementary Table S1. Program indicator definitions.

10.5334/gh.1595.s2 Supplementary Table S2. Glossary of terms and abbreviations.

## Data Availability

The manuscript is based on individual-level data collected through routine program monitoring of patient enrollment and follow-up. Because the dataset contains raw patient records, it is subject to strict confidentiality. For this reason, the dataset is not publicly available.
