# Supplementary material for: Bangladesh HEARTS: Lessons Learned from a National Hypertension Control Program, 2018–2026
Source: Glob Heart. 2026 Oct 21;21(1):74. doi: 10.5334/gh.1595 (PMC13693583; doi:10.5334/gh.1595)
Supplement: Supplementary file 1 [file gh-21-1-1595-s1.pdf]

Indicator	Definition
**Total registrations**	Total registered hypertensive patients
**BP controlled**	Patients with BP <140/90 at their last visit in the last three months
**BP not controlled**	Patients with BP ≥140/90 at their last visit in the last three months
**Overdue**	Patients who missed their scheduled follow-up visit by at least one day but no more than three months
**Missed visits**	Patients with no visits in the last three months
**Lost to follow-up**	Patients with no visits in the last one year
**Drug stock**	Days' supply of protocol antihypertensive medications

Supplementary Table S1: Program indicator definitions
