# Supplementary material for: Bangladesh HEARTS: Lessons Learned from a National Hypertension Control Program, 2018–2026
Source: Glob Heart. 2026 Oct 21;21(1):74. doi: 10.5334/gh.1595 (PMC13693583; doi:10.5334/gh.1595)
Supplement: Supplementary file 2 [file gh-21-1-1595-s2.pdf]

Supplementary table S2

**CB_HEARTS**	Community-Based HEARTS Intervention study.
**Civil Surgeon**	District manager (health services).
**Community Based Health Care (CBHC)**	CBHC is the organization that oversees all the operations of the Community Clinic.
**Community Clinic (CC)**	The Government of Bangladesh established CC to extend PHC to the doorsteps of villagers all over the country. CHCP is the service provider at the CCs. Bangladesh has established more than 13,000 community clinics to provide primary health care. Each CC cover about 6,000–10,000 people.
**Community Clinic Refilling**	Patient whose BP is under control for the last 3 visits in last 3 months can collect prescribed medication from the CC without visiting UHC. If BP is found not controlled the patient must visit the UHC for revaluation by a physician.
**Community Group (CG)**	For management of CC activities, there is 1 CG for each CC having 13–17 members. An elected UP member of that locality leads the group.
**Community Health care Provider (CHCP)**	Health service provider for the Community Clinic, 1 CHCP for each CC.

**Community Support Group (CSG)**	In the catchment area of each CC, there will be 3 CSGs comprising 13–17 members. The CSGs help CG in CC management along with making the community aware of the services available at CC and common health messages.
**Control Rate**	The hypertension control rate was defined as the ratio of individuals with systolic BP <140 mmHg and diastolic BP <90 mmHg among all individuals taking antihypertensive medication.
**DHIS2**	MIS-DGHS has established a web-based data collection system called District Health Information System (DHIS, version 2) to collect routine health data from the government health facilities of Bangladesh.
**Division**	The first-level administrative unit. There are 8 divisions of Bangladesh. Each division is named after the major city within its jurisdiction that also serves as the administrative headquarters of that division. Each division is further split into several districts which are then further subdivided into sub-districts or Upazilas.
**EDCL**	For drug procurement, stakeholders of the program are dependent on a single drug company – Essential Drug Company Limited (EDCL), operated by the Government of Bangladesh.
**Follow-up visit**	A visit made as a follow-up to an initial visit. Hypertension patients are typically asked to come back after 30 days for a routine follow-up visit.
**Missed visit**	Registered patients with no visit in the past 3 months addressed as missed visit.

**Screening**	Per Johns Hopkins Medicine, a screening test is done to detect potential health disorders or diseases in people who do not have any symptoms of disease. The goal is early detection and lifestyle changes or surveillance, to reduce the risk of disease, or to detect it early enough to treat it most effectively.
**Sub-district/Upazila**	An administrative region in Bangladesh, functioning as a sub-unit of a district (see Division).
**Union Parishad (UP)**	Union councils or Union parishads are the smallest rural administrative and local government units in Bangladesh. Each Union is made up of nine Wards. Usually one village is designated as a ward. There are 4,562 Unions in Bangladesh. A Union Council consists of a chairman and 12 members including three members exclusively reserved for women.
**Union Sub-Center (USC)**	A Union Sub-Center is a small rural public health facility at the union level (just below the upazila level) that provides basic healthcare, maternal and child services, family planning, immunizations, and referrals. USCs cover about 30,000–40,000 people in a Union.
**Upazila Health Complex (UHC)**	The public health care delivery systems at the Upazila/sub-district level. It is the primary public health care facility in Bangladesh. It provides outpatient care, primary health care, family planning services, and other preventive health care services. UHCs cover about 200,000–400,000 people in an Upazila.
**UH&FPO**	Facility manager of the UHC.

Supplementary Table S2. Glossary of Terms and Abbreviations
